# Development of a dual immunochromatographic test strip to detect E2 and E^rns^ antibodies against classical swine fever

**DOI:** 10.3389/fmicb.2024.1383976

**Published:** 2024-04-11

**Authors:** Loc Tan Huynh, Eun-Ju Sohn, Youngmin Park, Juhun Kim, Tomohiko Shimoda, Takahiro Hiono, Norikazu Isoda, Sung-Hee Hong, Ha-Na Lee, Yoshihiro Sakoda

**Affiliations:** ^1^Laboratory of Microbiology, Department of Disease Control, Faculty of Veterinary Medicine, Hokkaido University, Sapporo, Hokkaido, Japan; ^2^Faculty of Veterinary Medicine, College of Agriculture, Can Tho University, Can Tho, Vietnam; ^3^BioApplications, Inc., Pohang, Gyeongsangbuk, Republic of Korea; ^4^Nansei Livestock Hygiene Service Center, Matsuzaka, Mie, Japan; ^5^One Health Research Center, Hokkaido University, Sapporo, Hokkaido, Japan; ^6^International Collaboration Unit, International Institute for Zoonosis Control, Hokkaido University, Sapporo, Hokkaido, Japan; ^7^Institute for Vaccine Research and Development (HU-IVReD), Hokkaido University, Sapporo, Hokkaido, Japan; ^8^Celltrix Co., Ltd., Seongnam, Gyeonggi, Republic of Korea

**Keywords:** classical swine fever, immunochromatographic test strip, E2, E^rns^, antibody detection

## Abstract

**Background:**

It is essential to consider a practical antibody test to successfully implement marker vaccines and validate vaccination efficacy against classical swine fever virus (CSFV). The test should include a serological antibody assay, combined with a tool for differentiating infected from vaccinated animals (DIVA). The immunochromatographic test strip (ICS) has been exclusively designed for detecting CSFV E2 antibodies while lacking in detecting E^rns^ antibodies, which can be employed and satisfy DIVA strategy. This study developed a novel ICS for detecting CSFV E2/E^rns^ dual-antibody. The effectiveness of ICS in evaluating the DIVA capability of two novel chimeric pestivirus vaccine candidates was assessed.

**Methods:**

Recombinant E2 or E^rns^ protein was transiently expressed in the plant *benthamiana* using *Agrobacterium tumefaciens*. ICS was subsequently assembled, and goat anti-rabbit IgG and recombinant CSFV E2 or E^rns^ protein were plated onto the nitrocellulose membrane as control and test lines, respectively. The sensitivity and specificity of ICS were evaluated using sera with different neutralizing antibody titers or positive for antibodies against CSFV and other pestiviruses. The coincidence rates for detecting E2 and E^rns^ antibodies between ICS and commercial enzyme-linked immunosorbent assay (ELISA) kits were also computed. ICS performance for DIVA capability was evaluated using sera from pigs vaccinated with conventional vaccine or chimeric vaccine candidates.

**Results:**

E2 and E^rns^ proteins were successfully expressed in *N. benthamiana*-produced recombinant proteins. ICS demonstrated high sensitivity in identifying CSFV E2 and E^rns^ antibodies, even at the low neutralizing antibody titers. No cross-reactivity with antibodies from other pestiviruses was confirmed using ICS. There were high agreement rates of 93.0 and 96.5% between ICS and two commercial ELISA kits for E2 antibody testing. ICS also achieved strong coincidence rates of 92.9 and 89.3% with two ELISA kits for E^rns^ antibody detection. ICS confirmed the absence of CSFV E^rns^-specific antibodies in sera from pigs vaccinated with chimeric vaccine candidates.

**Conclusion:**

E2 and E^rns^ proteins derived from the plant showed great potential and can be used to engineer a CSFV E2/E^rns^ dual-antibody ICS. The ICS was also highly sensitive and specific for detecting CSFV E2 and E^rns^ antibodies. Significantly, ICS can fulfill the DIVA concept by incorporating chimeric vaccine candidates.

## 1 Introduction

Classical swine fever (CSF) is a highly infectious and fatal disease that has caused enormous mortality in domestic pigs and wild boars (Blome et al., 2017; Ganges et al., 2020). The disease is caused by CSF virus (CSFV), a positive-sense, enveloped single-stranded RNA virus belonging to the Pestivirus genus within the *Flaviviridae* family (Postel et al., 2021). The complete genome of CSFV is ~12.3 kb long, with a single large open reading frame encoding four structural proteins (C, E^rns^, E1, and E2) and eight nonstructural proteins (N^pro^, p7, NS2, NS3, NS4A, NS4B, NS5A, and NS5B) flanked by two untranslated regions (UTRs), 5′-UTR and 3′-UTR (Meyers and Thiel, 1996). After CSFV infection, an antibody response is elicited against envelope glycoproteins (E2 and E^rns^) and a nonstructural protein (NS3; König et al., 1995; Rau et al., 2006; Panyasing et al., 2018). Notably, envelope glycoprotein E2 is a prominent, protective antigen that can elicit neutralizing antibodies and is the primary target antigen of molecular and serological diagnostic assays used to assess vaccination effectiveness against CSFV (van Rijn et al., 1999; van Rijn, 2007). Furthermore, E^rns^ can also induce the neutralizing antibody production, and the antibody response to E^rns^ can be used as a marker to diagnose CSFV infection in pigs (König et al., 1995; Moormann et al., 2000; Lin et al., 2004).

In CSF-endemic countries, strategies involving biological safety precautions and routine vaccination using traditional live attenuated vaccines (LAVs) and subunit vaccines have been implemented to control and eradicate CSFV (Postel et al., 2018; Coronado et al., 2021). Nevertheless, failure of vaccination programs is an essential factor that impedes CSF control due to immunosuppression in pig herds caused by persistent infection with moderately or low virulent CSFV strains and the interference between vaccine types and maternally derived antibodies (MDA) in piglets (Coronado et al., 2021). These challenges contribute to reduced vaccine efficacy, resulting in sporadic outbreaks and CSF reemergence in vaccinated herds and leading to CSF-endemic disease (Pérez et al., 2012; Muñoz-González et al., 2015; Coronado et al., 2019). Hence, with the enforcement of improved surveillance systems and the advancement of efficient vaccines, developing efficient antibody detection systems is crucial for enhanced CSFV control. Considering this scenario, a suitable serological antibody assessment through extensive screening is needed to monitor the vaccination immunity of pigs against CSFV in practice (Blome et al., 2006; Schroeder et al., 2012).

Currently, the evaluation of antibody responses against CSFV is performed by serum neutralization test (SNT) and the enzyme-linked immunosorbent assay (ELISA) designed to detect antibody responses specific to the E2 or E^rns^ glycoprotein (Postel et al., 2018; Wang et al., 2020). Notably, detecting E^rns^ antibodies is a reliable diagnostic test for differentiating infected from vaccinated animals (DIVA) using marker vaccines that induce a protective immune response specific to CSFV E2 while showing an absence of specific antibodies to CSFV E^rns^ (Langedijk et al., 2001; Lin et al., 2005; Meyer et al., 2017). Given this context, traditional immunoassays, such as SNT or ELISA, can validate DIVA properties and vaccination efficacy of several marker vaccine candidates that have been recently generated, including chimeric pestiviruses CP_E2alf (Reimann et al., 2004) and Flc-LOM-BE^rns^ (Lim et al., 2019) or two novel chimeric viruses vGPE^−^/PAPeV E^rns^ and vGPE^−^/PhoPeV E^rns^ (Huynh et al., 2023) and E2 subunit vaccines (Gong et al., 2019; Park et al., 2021). However, these assays must be conducted in a laboratory setting and involve complex experimental procedures, significant time consumption, and substantial equipment and maintenance costs despite their high sensitivity and accuracy (Postel et al., 2018; Ganges et al., 2020; Wang et al., 2020). Therefore, a reliable and rapid DIVA assay should be considered to successfully implement marker vaccines and validate vaccination efficacy.

An immunochromatographic strip test (ICS) is the most rapid and reliable point-of-care diagnostic tool for prompt policy regarding effective disease control strategies (Manessis et al., 2022). ICS provides benefits compared to traditional immunoassays, such as affordability, rapidness, user-friendliness, and high specificity and sensitivity (Wang et al., 2020; Manessis et al., 2022). Previously, an ICS for CSFV antibody detection was developed based on the chimeric protein CnC2, generated from a specific region encoding E^rns^ (amino acids 109–160; assigned as Cn) and E2 (amino acids 1–176; assigned as C2) antigen epitopes of CSFV by the *Escherichia coli* expression system (Li et al., 2012). However, protein expression by the *E. coli* system can lead to protein misfolding, which may reduce antibody detection efficacy (Bai et al., 2022). Several ICS studies have been performed to improve CSFV E2 antibody detection by enhancing the protein expression system, such as the baculovirus expression system (Bai et al., 2022) or transgenic rice endosperm (Xu et al., 2022). However, ICS for CSFV E2 antibody detection can only be used to monitor vaccination efficacy and early detection of antibody responses, whereas the DIVA test for detecting E^rns^ antibody is lacking.

Plant-based production systems are promising platforms for recombinant protein production due to their numerous advantages over traditional expression systems (Qiu et al., 2014; Ma et al., 2015). Among these plant systems, *Nicotiana benthamiana*, a close relative of tobacco, has gained widespread attention for its exceptional suitability for recombinant protein expression. *N. benthamiana* offers several advantages for recombinant protein production (Ward et al., 2020; Hager et al., 2022; Kallolimath et al., 2023) and is fast-growing, easily cultivated, and amenable to genetic manipulation, making it an attractive host for protein expression studies. Furthermore, *N. benthamiana* also possesses a robust protein folding and posttranslational modification machinery, allowing for the production of complex, biologically active proteins. A key advantage of *N. benthamiana* is its ability to produce glycosylated proteins with mammalian-like glycosylation patterns which is particularly advantageous for producing therapeutic proteins because proper glycosylation is often critical for protein stability, functionality, and immunogenicity (Strasser, 2023). Therefore, *N. benthamiana*-based expression system highlight their advantages, applications, and prospects.

In a previous study, two chimeric pestiviruses, namely vGPE^−^/PAPeV E^rns^ and vGPE^−^/PhoPeV E^rns^, were generated based on the backbone of a CSFV LAV strain combined with the glycoprotein E^rns^ distantly related to CSFV (Huynh et al., 2023). These two chimeric viruses were proposed as promising marker vaccine candidates, suggesting a feasible approach for establishing an enhanced serological negative marker in DIVA vaccine development for CSF. However, DIVA capability was still not assessed for these chimeric vaccine candidates.

Thus, in this study, a novel ICS was developed to rapidly and reliably detect CSFV E2 and E^rns^ antibodies. Recombinant E2 and E^rns^ proteins were transiently expressed in *N. benthamiana* using *Agrobacterium tumefaciens*. Subsequently, ICS was constructed to detect CSFV E2 and E^rns^ antibodies. In addition, ICS performance was compared to commercial ELISA kits for CSFV E2 or E^rns^ antibody detection. Furthermore, a panel of serum samples derived from pigs vaccinated with chimeric vaccine candidates was utilized, incorporating ICS to evaluate DIVA capability.

## 2 Materials and methods

### 2.1 Cloning of CSFV E2 and E^rns^ genes

The full-length E2 (derived from CSFV Alfort A19 strain) and E^rns^ (derived from LOM strain) encoding sequences were obtained from the National Center for Biotechnology Information (NCBI) database (GenBank accession numbers AAB50409.1 and ABD83960, respectively) and optimized for expression in *N. benthamiana*^1^ before gene synthesis. For CSFV E2 expression, genes encoding the signal peptide of BiP (immunoglobulin heavy-chain binding protein) with a length of 271 nucleotides (nt) -the endoplasmic reticulum (ER) chaperone, full-length CSFV E2 sequence (1,023 nt), linker (CAGATTACGACATTCCAACAACTGATGCA), 6 histidines, cellulose binding domain (CBD; 454 nt) derived from xynA of *Clostridium stercorarium*, and ER retention signal (His-Asp-Glu-Leu) were fused sequentially and then cloned into the pCAMBIA1300 vector (Park et al., 2019) harboring the 35S promoter and heat shock protein terminator. The expected size of the fusion protein was approximately 58 kDa. For E^rns^ expression, genes encoding the signal peptide of chaperone binding protein (251 nt), full-length CSFV E^rns^ (681 nt), linker (GGTGGGGGAGGCAGT), 10 histidines, and ER retention signal (His-Asp-Glu-Leu) were fused sequentially and cloned into a pTEX vector harboring the MacT promoter and RD29B terminator. The expected size of the fusion protein was nearly 27.7 kDa. The nucleotide sequence was verified by sequencing (Bioneer, Daejeon, Republic of Korea).

### 2.2 Transient expression of recombinant CSFV E2 and E^rns^

The plasmid for expressing recombinant CSFV E2 or E^rns^ was transformed into the *A. tumefaciens* GV3101 strain (Lifeasible, Shirley, NY, United States) by electroporation. Transformed *A. tumefaciens* were grown for 16 h in a 5 mL yeast extract peptone (YEP) liquid medium supplemented with 50 mg/L kanamycin and 25 mg/L rifampicin. Next, 1 mL of cultured bacteria was inoculated into 1 L of fresh YEP medium and cultured for a further 16 h at 28°C. Bacteria were pelleted by centrifugation at 7,341 × g for 5 min at 4°C and resuspended at the desired concentration (determined by measuring OD_600_) in a solution consisting of 10 mM 2-(N-morpholino) ethane sulfonic acid (MES) (Duksan, Seoul, Republic of Korea), 10 mM magnesium chloride (Sigma-Aldrich, St. Louis, MO, United States), and 100 μM acetosyringone (Sigma-Aldrich) at pH 5.6. Agroinfiltration was performed under a vacuum. After 4 days, the leaves were harvested for protein purification.

### 2.3 Purification of CSFV E2 and E^rns^ protein

In the purification of E2 protein, the leaves were crushed and incubated for 30 min in an extraction buffer 1 (EB 1) consisting of 50 mM sodium phosphate (pH 8.0), 300 mM sodium chloride (NaCl), 10 mM imidazole, 0.5% (v/v) Triton X-100, 50 mM glycine, 100 mM sodium sulfite, 50 mM ascorbic acid, and 1.5% polyvinylpolypyrrolidone. With the purification of E^rns^ protein, an extraction buffer 2 (EB 2) consisting of 50 mM Tris-Cl (pH 8.5), 300 mM NaCl, 50 mM imidazole, 0.3% (v/v) Triton X-100, 100 mM glycine, 100 mM sodium sulfite, 1 mM phenyl methyl sulfonyl fluoride, and 1.5% polyvinylpolypyrrolidone was used. After centrifugation, the supernatant from EB 1 or EB 2 was mixed with His60 Ni Superflow Resin (Takara, Kusatsu, Japan) for 1 h at 4°C, and the bound proteins were eluted with 50 mM sodium phosphate (pH 8.0), 300 mM NaCl, and 250 mM imidazole for E2 protein or 300 mM imidazole for E^rns^ protein purification. The eluates were adjusted with 50 mM Tris-Cl (pH 7.2) and 150 mM NaCl. The purified proteins were treated with β-mercaptoethanol, assessed by 10% sodium dodecyl sulfate-polyacrylamide gel electrophoresis (SDS-PAGE), and analyzed by Western blotting with an anti-His antibody (BioLegend, San Diego, CA, United States), anti-LOM vaccinated serum, and anti-E2 subunit vaccinated serum.

### 2.4 Manufacture of the prototype immunochromatographic test strip

#### 2.4.1 Colloidal gold solution preparation

The colloidal gold solution (in-house) was prepared by adding 90 mL of distilled water into a triangular flask and heating it to 100°C while stirring on a hot stirrer plate (DAIHAN Scientific, Wonju, Republic of Korea). Once the temperature reached 100°C, 1% (w/v) gold chloride trihydrate (10 mL) was added and dissolved. After the gold chloride trihydrate solution was completely dissolved, 1 mL of 1% sodium citrate tribasic dehydrate was added and dissolved for 10 min at room temperature until the color of the reaction solution changed from yellow to the final red color. The resulting colloidal gold solution was diluted with distilled water, and the absorbance was measured using a UV-1650PC spectrophotometer (Shimazu, Tokyo, Japan).

#### 2.4.2 Conjugation of the CSFV E2 and E^rns^ and pretreatment of the conjugate pad

For the conjugation of recombinant proteins to the colloidal gold, 50 mL of colloidal gold solution was adjusted to pH 7.2 using 0.1 M potassium carbonate. Next, the recombinant E2 or E^rns^ solution with a concentration of 50 μg/mL in phosphate-buffered saline (PBS) was slowly added dropwise, one drop at a time, into the colloidal gold solution, and the mixture was stirred for 30 min on a stirrer. Then, 10% (w/v) bovine serum albumin (BSA; 5 mL) was added and stirred for an additional 30 min. The resulting conjugation solution was centrifuged to separate the upper layer for 30 min at 10,000 rpm, and the pellet was suspended in a solution containing 1% (w/v) BSA in PBS. Subsequently, to pretreat the conjugate pad with glass fiber material, a solution containing PBS, 3% sucrose, 0.1% sodium azide, 1% (w/v) BSA, recombinant E2 or E^rns^ conjugate (1.1 μg/kit), and rabbit IgG (in-house) conjugate (0.88 μg/kit), was evenly absorbed into the glass fiber material of the conjugate pad. The conjugate pad was dried for >4 h in an environment with a relative humidity of <20%.

#### 2.4.3 Fixation of the control and test lines and kit assembly

The control and test lines were fixed using nitrocellulose membranes (Adventec, Irvine, CA, United States). The control line was coated with 1 mg/mL goat anti-rabbit IgG (Arista Biologicals, Allentown, PA, United States), and the test line was coated with each recombinant protein (E2 or E^rns^) at 1 mg/mL. The membranes were dispensed using a dispenser, Matrix 1600 (Kinematic Automation, Sonora, CA, United States), at a rate of 1 μL/cm and dried for >4 h in an environment with a relative humidity of <20%. In the next step, the kit was assembled by attaching the membrane coated with the antigen onto the adhesive card, followed by the conjugate pad and sample pad overlaid to overlap at the bottom. The assembled kit was cut to a width of 4 mm and inserted into a plastic device.

### 2.5 Swine serum samples

#### 2.5.1 Samples for evaluating the sensitivity and specificity of immunochromatographic test strip

ICS sensitivity was assessed using 23 serum samples with varying SNT titers obtained from a previous study (Oh et al., 2021), sourced from two distinct groups: LOM-vaccinated pigs (*n* = 10) and nonvaccinated pigs with MDA (*n* = 13). Furthermore, ICS specificity was evaluated using swine serum samples (*n* = 5) that were positive for antibodies against CSFV, bovine viral diarrhea virus (BVDV), or border disease virus (BDV) from a previous study (Sakoda et al., 2012). Additionally, CSFV antibody-negative serum samples (*n* = 50) from naïve pigs were included in the specificity analysis. Furthermore, the serum samples (*n* = 9) were obtained from pigs infected with African swine fever virus (ASFV), porcine circovirus 2 (PCV2), and porcine reproductive and respiratory syndrome virus (PRRSV) that were confirmed positive using commercial ELISA kits: ID Screen® African Swine Fever Indirect (Innovative Diagnostics, Grabels, France), PCV2 Antibody Test Kit (BioChek, Scarborough, ME, United States), and IDEXX PRRS X3 Ab Test (IDEXX, Westbrook, ME, United States), respectively.

#### 2.5.2 Sera for determining the coincidence rates between the immunochromatographic test strip and commercial enzyme-linked immunosorbent assay kits

Six CSFV-negative piglets were purchased from a CSFV-negative farm located in Jeju Island, Republic of Korea, and confirmed by screening with an ELISA assay for E2 antibody detection. Piglets were injected intramuscularly twice on the neck at 40 days of age [0 day postvaccination (dpv)] and 60 days (20 dpv) after birth using the CSFV E2 subunit vaccine (BioApplications, Pohang, Republic of Korea), which was developed and evaluated in previous studies (Park et al., 2020, 2021). Sera were collected at 40 days of age (before vaccination), 60 days (20 days after the first vaccination), and 80 days (40 days after the first vaccination). Eighteen serum samples were used to compare the agreement rate between ICS and ELISA kits.

Recently, the LOM strain initially employed as a vaccine strain, was introduced to Jeju Island, where is known for its no-vaccination policy against CSFV. Unfortunately, the CSFV has started spreading from sows to piglets and has undergone mutations during its transmission. Consequently, the Government is promoting a CSFV marker vaccine policy to combat and eliminate CSF (Oh et al., 2021). To gather clinical sera for analysis, 57 serum samples were randomly obtained from CSFV-positive and-negative farms, subsequently determined to be positive and-negative for E2 and E^rns^ antibodies by ELISA kits, respectively. These samples were used to evaluate the coincidence rates of ICS with ELISA kits for detecting E^rns^ and E2 antibodies.

The definition and way of calculation of total coincidence rates and positive coincidence rates are as followed (Pan et al., 2019): total coincidence rates = 100% × [(both positive of ICS and ELISA kit + both negative of ICS and ELISA kit)/total samples]; compared to ELISA kit, the positive coincidence rates of ICS = 100% × [both positive of ICS and ELISA kit/(both positive of ICS and ELISA kit + ICS positive but ELISA kit negative)].

The performance of ICS was compared with commercial ELISA kits in terms of relative sensitivity and specificity, as followed (Zhong et al., 2021): relative sensitivity = 100% × [(the number of true positive samples)/(the number of true positive samples + the number of false negative samples)]; relative specificity = 100% × [(the number of true negative samples)/(the number of true negative samples + the number of false positive samples)].

#### 2.5.3 Evaluation of the immunochromatographic test strip using serum samples from pigs vaccinated with chimeric virus and commercial live attenuated-vaccine

Serum samples from pigs vaccinated with novel chimeric vaccine candidates in a previous study (Huynh et al., 2023) were used to evaluate DIVA properties using a novel ICS. In detail, 52 serum samples from 26 pigs vaccinated with each chimeric virus candidate (vGPE^−^/PAPeV E^rns^ or vGPE^−^/PhoPeV E^rns^) or vGPE^−^ derived from a conventional LAV were evaluated at 0 and 21 dpv. Additionally, 45 serum samples from 15 pigs were collected at 0, 7 dpv and 14 days postchallenge (dpc) in challenge studies that were also assessed. In these trials, each chimeric virus candidate or vGPE^−^ was independently administered to pigs 7 days before exposure to the moderately virulent CSFV vALD-A76 strain. Subsequently, continuous monitoring was conducted for 14 days. Furthermore, the vaccination efficacy of piglets with MDA (*n* = 6) immunized with a commercial conventional CSFV GPE^−^ LAV strain was also investigated, involving the collection of 24 serum samples at different time intervals (0, 45, and 90 dpv) and before slaughtering.

### 2.6 Enzyme-linked immunosorbent assay

The assay was performed using the PrioCHECK CSFV E^rns^ Antibody ELISA Kit (Thermo Fisher Scientific, Waltham, MA, United States), CSFV Antibody Test Kit (IDEXX, Westbrook, ME, United States), CSFV Antibody B-ELISA (Bionote, Gyenggi-do, Republic of Korea), and Pigtype CSFV E^rns^ Antibody (Indical Bioscience, Leipzig, Germany). The assay procedures were performed according to the manufacturers’ protocol. The absorbance of the solution was subsequently read at 450 nm on a Muliskan ELISA reader (Thermo Fisher Scientific).

### 2.7 Serum neutralization test

The test was performed using a luciferase-based assay, as described previously (Tetsuo et al., 2020). The determination of neutralizing antibody titers in serum samples derived from pigs vaccinated with each chimeric virus candidate, vGPE^−^, or commercial conventional CSFV GPE^−^ LAV strain was accomplished through a luciferase assay employing the Nano-Glo HiBiT lytic detection system (Promega, Madison, WI, USA) and POWERSCAN®4 (Agilent Technologies International Japan Ltd., Tokyo, Japan).

The neutralizing antibodies against CSFV in serum samples from a previous study (Oh et al., 2021) were tested using a neutralizing peroxidase-linked assay described previously (Park et al., 2020). In brief, equal volumes of serially diluted serum and 200 TCID_50_ of the CSFV LOM LAV strain were mixed well and incubated at 37°C in 5% CO_2_ for 1 h. The mixture and 100 μL cloned porcine kidney cell suspension were incubated in 96-well plates at 37°C in 5% CO_2_ for 72 h. Cells were fixed with 80% acetone and dried at 37°C, followed by staining with commercial anti-LOM antibody as the primary antibody for immunoperoxidase staining (Park et al., 2020). The SNT titers represent the reciprocal of the highest dilution that achieved 50% neutralization.

## 3 Results

### 3.1 Expression and purification of recombinant CSFV E2 and E^rns^ proteins

**Figure 1:**
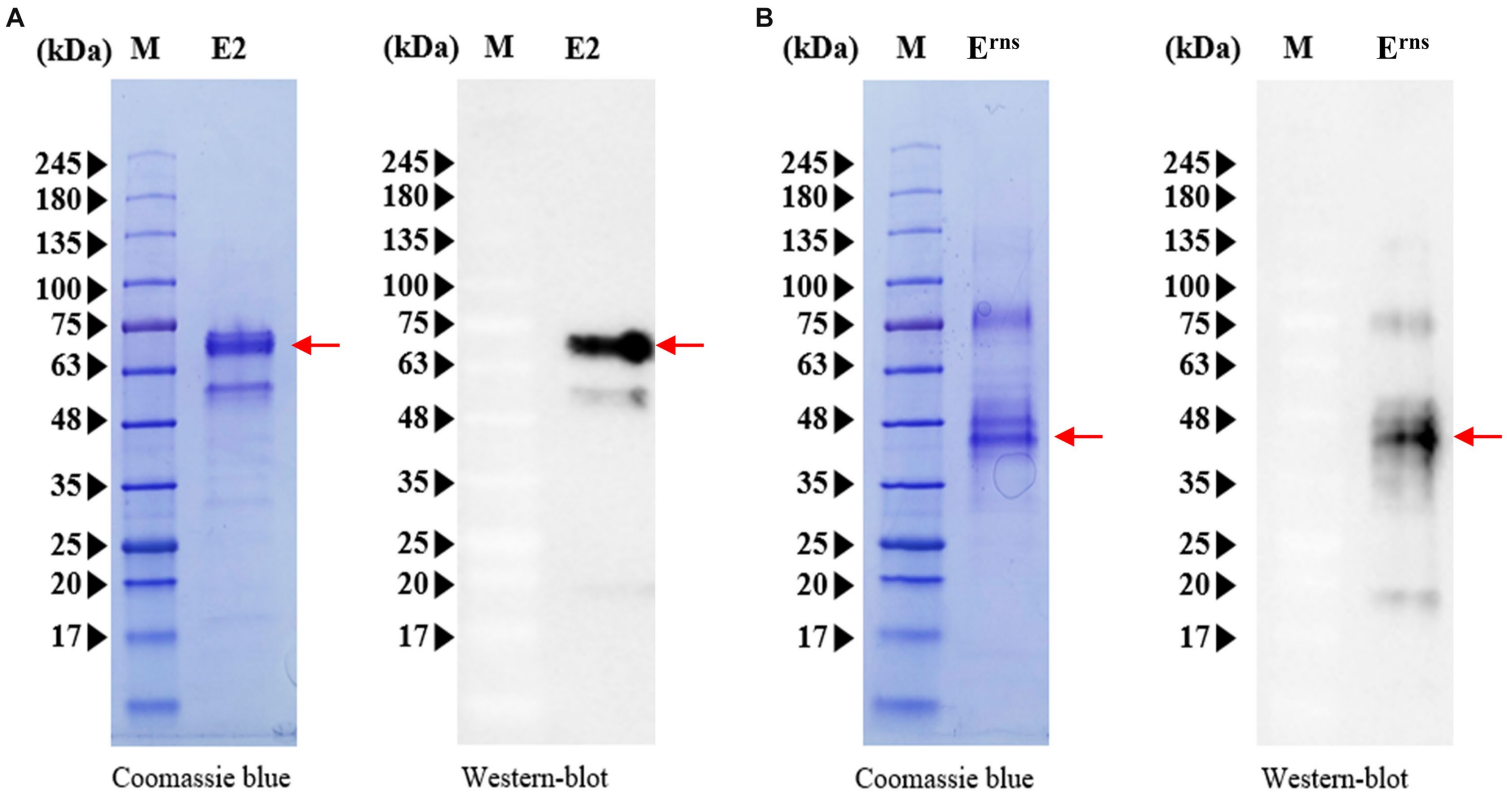
Expression and characterization of CSFV E2 and E^rns^ proteins. **(A)** Purified E2 protein was subjected to Western blotting (right panels) using an anti-His monoclonal antibody, and gels were stained with Coomassie brilliant blue (left panels) after SDS-PAGE analysis. **(B)** Western blot analysis of purified E^rns^ protein (right panels) using an anti-His monoclonal antibody and SDS-PAGE analysis (left panels). M, protein size marker. Red arrows represent the expression of E2 or E^rns^ protein.

The sequences encoding CSFV E2 and E^rns^ proteins were cloned into the *A. tumefaciens* GV3101 strain, and either the E2 or E^rns^ recombinant proteins were expressed in *N. benthamiana* plants. The harvested leaves were crushed and subjected to His60 Ni Superflow Resin for protein purification. The purified proteins were then assessed by Western blotting and SDS-PAGE. As illustrated in Figure 1, the glycosylated forms of recombinant E2 and E^rns^ proteins were confirmed by anti-His antibody. The recombinant E2 protein exhibited a visible band of ~69 kDa, whereas the recombinant E^rns^ protein appeared at ~45 kDa under denaturing conditions (Figure 1). Moreover, verification of the aforementioned findings was achieved by anti-LOM vaccinated serum (demonstrating positivity for E2 and E^rns^) and anti-E2 subunit vaccinated serum (displaying positivity exclusively for E2) for the purpose of validating protein expression via Western blot analysis (Supplementary Figure S1). Although the bands appeared to be diffuse in the analysis of E^rns^ protein, the deglycosylation with glycosidase PNGase F resulted in a single intense band, and the molecular weights of E^rns^ were approximately 28 kDa, which corresponded to its calculated molecular weights (Supplementary Figure S1D). Moreover, the intense band exceeded the anticipated molecular weights of recombinant E2 (58 kDa) or E^rns^ (28 kDa) fusion protein, suggesting that two proteins were extensively glycosylated, particularly with *N*-glycosylation contributing to sizes larger than anticipated that were observed when expressed in plants (Park et al., 2019, 2020). Taken together, the different glycosylation levels of each protein indicate that these two proteins are properly glycosylated and can serve as suitable antigens for further experiments.

### 3.2 Conjugation of recombinant proteins with the gold nanoparticles and implementation of the working principle of the developed CSFV E2 and E^rns^ detection kit

**Figure 2:**
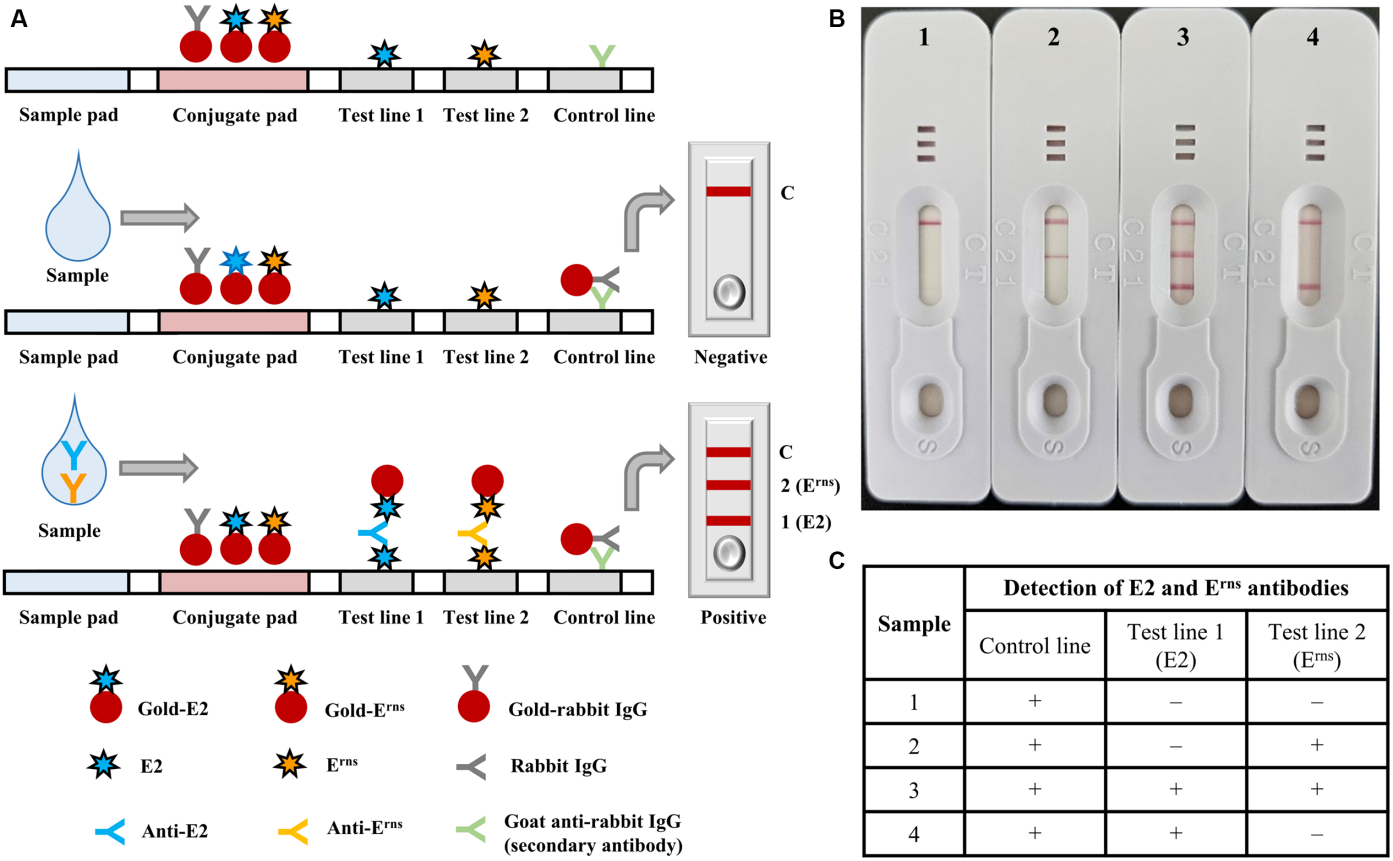
Diagrammatic illustration and implementation example of the immunochromatographic test strip. **(A)** Structural composition of the immunochromatographic test strip, including sample and conjugate pads, test lines 1 and 2, and control line; negative and positive results were also indicated. **(B)** The implementation of representative serum samples using the immunochromatographic test strip 1 (negative control), 2 (positive with E^rns^), 3 (positive with both E2 and E^rns^), and 4 (positive with E2), in which the serum samples were diluted with dilution buffer at a ratio of 1:1. **(C)** Representative results of each sample were summarized in the table.

The optimal protein concentration for creating the E2 or E^rns^ conjugate was 1.25 O.D., using a spectrophotometer at a wavelength of 540 nm and a particle size of 40 nm. The ICS assembly is illustrated in Figure 2. The assembled ICS comprised a membrane coated with the antigen onto the adhesive card, followed by a conjugate pad and sample pad overlaid to overlap at the bottom. Nitrocellulose membranes were used to fix the control line (C) and test line (T) membranes in the ICS. Specifically, the control line was plated with a second antibody (goat anti-rabbit IgG). Meanwhile, the test line was plated with recombinant E2 and E^rns^ proteins corresponding to numbers 1 and 2 in the ICS (Figure 2A). In accordance with usage principles, serum samples from pigs were loaded on the application pad and diffused with gold-rabbit IgG and gold-E2 or E^rns^ on the conjugate pad. For the control line, gold-rabbit IgG is bound to anti-rabbit IgG antibodies and accumulated. The anti-E2 or E^rns^ antibody in the serum sample is bound and diffused with gold-E2 or E^rns^ on the conjugate pad, and the complex bound to the E2 or E^rns^ protein on the test line. Consequently, a red color appeared on the test line for the positive samples due to the accumulated complex. In contrast, the test line will not display red color for the negative samples that did not have anti-E2 or E^rns^ antibodies (Figures 2B,C).

### 3.3 Specificity and sensitivity of the immunochromatographic test strip

The sensitivity of ICS was evaluated using various serum samples positive for CSFV with different SNT titers. Additionally, the specificity of ICS was assessed using sera from CSFV-and pestivirus-inoculated pigs, and sera from naïve pigs. Twenty-three serum samples ranging from the lowest SNT titer of 4 to the highest SNT titer of 2,048 were subjected to ICS testing. The results in Table 1 demonstrated that ICS effectively detected antibodies against CSFV E2 and E^rns^ in all samples. The E2 and E^rns^ antibodies were detected at the lowest SNT titers of 16 and 4, respectively. These findings demonstrated the high sensitivity of the ICS for detecting E2 and E^rns^ antibodies, with particular sensitivity for CSFV E^rns^ antibody detection. Furthermore, the specificity of ICS for CSFV E2 and E^rns^ antibody detection was assessed. Table 2 revealed that two CSFV-positive serum samples were also confirmed for detecting both E2 and E^rns^ antibodies using ICS. In contrast, other pestivirus-positive serum samples (*n* = 3), including BVDV strains Nose (BVDV genotype 1a) and KZ-91-NCP (BVDV genotype 2a), BDV, and CSFV-negative sera from naïve pigs (*n* = 50) tested negative for E2 and E^rns^ antibodies. Furthermore, additional serum samples (*n* = 9), consisting of ASFV (*n* = 3), PCV2 (*n* = 3), and PRRSV (*n* = 3) were also confirmed as negative. Data suggested that the ICS exhibited no cross-reactivity with antibodies from other swine pestiviruses and related swine viral diseases.

**Table 1:** Sensitivity of immunochromatographic test strip for detecting E2 and E^rns^ antibodies using various swine serum samples with different neutralizing antibody titers.

Neutralizing antibody titers	Antibody detection				Total
	E2		E^rns^		
	Positive	Negative	Positive	Negative	
4	0	1	1	0	1
16	1	0	1	0	1
32	7	0	7	0	7
64	3	0	3	0	3
128	2	0	2	0	2
256	3	0	3	0	3
512	2	0	2	0	2
1,024	2	0	2	0	2
2,048	2	0	2	0	2

**Table 2:** Specificity of the immunochromatographic test strip for detecting E2 and E^rns^ antibodies in reference swine serum samples.

Serum samples	Days postinoculation	Antibody detection				Total
		E2		E^rns^		
		Positive	Negative	Positive	Negative	
CSFV GPE^—^positive*	33	1	0	1	0	1
CSFV Kanagawa/74-positive*	32	1	0	1	0	1
BVDV Nose-positive*	32	0	1	0	1	1
BVDV KZ-91 NCP-positive*	33	0	1	0	1	1
BDV 87/6-positive*	32	0	1	0	1	1
CSFV-negative	0	0	50	0	50	50
ASFV	7	0	3	0	3	3
PCV2	21	0	3	0	3	3
PRRSV	14	0	3	0	3	3

*Serum samples were obtained from the source as outlined previously (Sakoda et al., 2012); ASFV, African swine fever virus; CSFV, classical swine fever; BVDV, bovine viral diarrhea virus; BDV, border disease virus; PCV2, porcine circovirus 2; PRRSV, porcine reproductive and respiratory syndrome virus.

### 3.4 CSFV E2 and E^rns^ antibodies detection using immunochromatographic test strip and commercial enzyme-linked immunosorbent assay kits

The correlation between ICS and ELISA kits was then examined. Fifty-seven serum samples were randomly obtained from CSFV-positive farms positive for E2 antibody using ELISA kits (Bionote and IDEXX ELISA kits) and CSFV-negative farms that were tested to be negative for E^rns^ antibody using two commercial ELISA kits (Indical Bioscience and Thermo Fisher Scientific ELISA kits). However, one serum sample was in poor condition during analysis and was positive for E2 antibody but could not be pinpointed as the band for E^rns^ antibody detection. Therefore, the total number of E2 and E^rns^ antibody analysis samples was 57 and 56, respectively. The results in Table 3 indicated that E2 positive proportions of the ICS corresponding to the Bionote and IDEXX ELISA kits were 100% (34 of 34) and 94.1% (32 of 34), respectively. The sensitivity and specificity of ICS corresponding to the Bionote ELISA kits were 89.5% (34 of 38) and 100% (19 of 19), respectively. In addition, the high sensitivity and specificity of ICS corresponding to the IDEXX ELISA kits were 100% (32 of 32) and 92.0% (23 of 25), respectively. A high agreement rate of ICS was determined to be 93.0% (53 of 57) and 96.5% (55 of 57), corresponding to the Bionote and IDEXX ELISA kits, respectively. In Table 4, the E^rns^ positive percentages of ICS corresponding to the Indical Bioscience and Thermo Fisher Scientific ELISA kits were 86.2% (25 of 29) and 79.3% (23 of 29), respectively. ICS sensitivity for E^rns^ antibody detection corresponding to the ELISA kits was 100% for the Indical Bioscience ELISA kits (25 of 25) and the Thermo Fisher Scientific ELISA kits (23 of 23). ICS showed high specificity for E^rns^ detection at 87.1%, corresponding to the Indical Bioscience ELISA kits (27 of 31) and 81.8%, conforming to the Thermo Fisher Scientific ELISA kits (27 of 33). The agreement rates of ICS with the Indical Bioscience and Thermo Fisher Scientific ELISA kits were 92.9% (52 of 56) and 89.3% (50 of 56), respectively.

**Table 3:** Correlation between the immunochromatographic test strip and the two commercial ELISA kits for detecting CSFV E2 antibody.

ICS		Com 1		Com 2		Total
		Positive	Negative	Positive	Negative	
E2	Positive	34	0	32	2	34
	Negative	4	19	0	23	23
	Total	38	19	32	25	57

ICS, immunochromatographic test strip; Com 1, commercial Bionote ELISA kit; Com 2, commercial IDEXX ELISA kit.

**Table 4:** Correlation between the immunochromatographic test strip and the two commercial ELISA kits for detecting CSFV E^rns^ antibody.

ICS		Com 3		Com 4		Total
		Positive	Negative	Positive	Negative	
E^rns^	Positive	25	4	23	6	29
	Negative	0	27	0	27	27
	Total	25	31	23	33	56

ICS, immunochromatographic test strip; Com 3, commercial Indical Bioscience ELISA kit; Com 4, commercial Thermo Fisher Scientific ELISA kit.

ICS also strongly agreed with commercial ELISA kits in detecting E2 and E^rns^ antibody responses to the subunit CSFV vaccine. This was observed in serum samples collected from pigs immunized twice with the CSFV E2 subunit vaccine at 40 and 60 days of age. As presented in Supplementary Table S1, all swine sera tested negative for the E^rns^ antibody using commercial Bionote and IDEXX ELISA kits and ICS. The E2 antibody detection results using ICS aligned with those obtained from the commercial Bionote and IDEXX ELISA kits using serum samples collected at 40 dpv. This confirmation suggested that the ICS could detect the E2 antibody at a late stage in subunit-vaccinated pigs (Supplementary Table S1). Taken together, ICS was coincident at a high rate with the commercial ELISA kits used for detecting E2 and E^rns^ antibodies, which would emerge as a superior choice.

### 3.5 Diagnostic capability of the immunochromatographic test strip in differentiating infected from vaccinated animals using sera of pigs vaccinated with the chimeric vaccine candidates vGPE^−^/PAPeV E^rns^ and vGPE^−^/PhoPeV E^rns^

The swine serum samples obtained from two independent experiments from a previous study (Huynh et al., 2023) were used to assess the applicability of ICS for DIVA. In detail, serum samples from 26 pigs in the optimal infectious dose experiments were tested at 0 and 21 dpv using the ICS (Table 5). As shown in Table 5, all serum samples were negative for E2 and E^rns^ antibodies at 0 dpv, consistent with SNT results. In addition, CSFV E2 neutralizing antibody was detected in the serum samples at 21 dpv of pigs immunized with vGPE^−^/PAPeV E^rns^ (6 of 9) or vGPE^−^/PhoPeV E^rns^ (4 of 4). At the same time, there were undetectable against E^rns^ antibody in all pigs vaccinated with each chimeric virus. Conversely, the E^rns^ antibody was detected in almost all serum samples from pigs vaccinated with conventional vGPE^−^ strain, whereas only three of nine swine serum samples showed a positive result for the E2 antibody. Likewise, serum samples taken at different stages postvaccination from pigs vaccinated with commercial live attenuated GPE^−^ vaccine were used to evaluate the performance of ICS. Most serum samples exhibiting high SNT titers showed a high positive rate for either E2 or E^rns^ antibody or both, whereas samples with low SNT titers or a vaccination period <45 days were negative using ICS. Here, the anti-CSFV E2 antibody was detected in 18 of 24 serum samples, whereas 14 of 24 tested sera were positive for E^rns^ antibody, primarily observed between 90 dpv and before slaughtering (Supplementary Table S2). Furthermore, antibody responses to E2 and E^rns^ were assessed using serum samples collected at 0 and 7 dpv, and 14 dpc (or 21 dpv) in challenge studies, in which each chimeric virus candidate (vGPE^−^/PAPeV E^rns^ or vGPE^−^/PhoPeV E^rns^) or a conventional LAV vGPE^−^ was independently administered 7 days before challenge with the moderately virulent CSFV vALD-A76 strain and continuously monitored for 14 dpc. As presented in Table 6, the results showed that the E2 and E^rns^ antibodies were undetected in all serum samples at 0 and 7 dpv. However, an increasing tendency of the E2 antibody was confirmed with positive results in several serum samples from pigs vaccinated with each chimeric virus at 14 dpc, whereas the E^rns^ antibody was negative for all serum samples, as expected. It is crucial to notice that the E^rns^ antibody was detected only in all pigs immunized with conventional LAV and the control groups (unvaccinated pigs), whereas it was negative for those vaccinated with each chimeric virus mentioned above at 14 dpc. The results of E2 antibody detection in sera from unvaccinated pigs were negative in all serum samples at 14 dpc. In contrast, the E2 antibody against CSFV vGPE^−^ required a longer time to reach the detection threshold on the ICS than the SNT test.

**Table 5:** Detection of E2 and E^rns^ antibody responses from serum samples in pigs inoculated with vGPE^−^, vGPE^−^/PAPeV E^rns^, or vGPE^−^/PhoPeV E^rns^ at different doses of TCID_50_ using the immunochromatographic test strip and serum neutralization test.

Virus	Inoculation dose (TCID_50_)	Pig ID*	0 dpv			21 dpv		
			E2^**^	E^rns**^	SNT	E2^**^	E^rns**^	SNT
vGPE^−^/PAPeV E^rns^	10^3.0^	#312	−	−	<1	−	−	−
		#313	−	−	<1	+	−	1
		#314	−	−	<1	−	−	−
	10^4.0^	#315	−	−	<1	+	−	8
		#316	−	−	<1	−	−	1
		#317	−	−	<1	+	−	2
	10^5.0^	#318	−	−	<1	+	−	8
		#319	−	−	<1	+	−	32
		#320	−	−	<1	+	−	4
vGPE^−^/PhoPeV E^rns^	10^3.0^	#354	−	−	<1	+	−	2
		#355	−	−	<1	−	−	<1
		#356	−	−	<1	+	−	2
	10^4.0^	#357	−	−	<1	−	−	1
		#358	−	−	<1	−	−	1
		#359	−	−	<1	−	−	1
	10^5.0^	#360	−	−	<1	+	−	8
		#362	−	−	<1	+	−	32
vGPE^−^	10^3.0^	#303	−	−	<1	−	+	1
		#304	−	−	<1	−	−	<1
		#305	−	−	<1	−	+	4
	10^4.0^	#306	−	−	<1	−	+	4
		#307	−	−	<1	−	+	4
		#308	−	−	<1	+	+	8
	10^5.0^	#309	−	−	<1	+	+	4
		#310	−	−	<1	−	−	1
		#311	−	−	<1	+	+	8

*Serum samples were obtained from the source as outlined previously (Huynh et al., 2023); **antibody detection using immunochromatographic test strip; dpv, days postvaccination; SNT, serum neutralization test; −, negative; +, positive.

**Table 6:** Detection of E2 and E^rns^ antibody responses from serum samples in pigs vaccinated with vGPE^−^, vGPE^−^/PAPeV E^rns^, or vGPE^−^/PhoPeV E^rns^ in the challenge studies using the immunochromatographic test strip and serum neutralization test.

Virus	Pig ID*	0 dpv			7 dpv			14 dpc		
		E2^**^	E^rns**^	SNT	E2^**^	E^rns**^	SNT	E2^**^	E^rns**^	SNT
vGPE^−^/PAPeV E^rns^	#353	−	−	<1	−	−	<1	−	−	2
	#352	−	−	<1	−	−	<1	+	−	16
	#351	−	−	<1	−	−	<1	−	−	4
vGPE^−^/PhoPeV E^rns^	#373	−	−	<1	−	−	<1	+	−	4
	#372	−	−	<1	−	−	<1	+	−	8
	#371	−	−	<1	−	−	<1	+	−	4
vGPE^−^	#350	−	−	<1	−	−	<1	−	+	4
	#349	−	−	<1	−	−	<1	+	+	16
	#348	−	−	<1	−	−	<1	−	+	8
Nonvaccination	#347	−	−	<1	−	−	<1	−	+	<1
	#346	−	−	<1	−	−	<1	−	+	<1
	#345	−	−	<1	−	−	<1	−	+	<1
	#370	−	−	<1	−	−	<1	−	+	<1
	#369	−	−	<1	−	−	<1	−	+	<1
	#368	−	−	<1	−	−	<1	−	+	<1

*Serum samples were obtained from the source as outlined previously (Huynh et al., 2023). Briefly, pigs were immunized at a dose of 10^4.0^ TCID_50_ with each chimeric vaccine candidate, vGPE^−^, and unvaccinated pigs. All pigs were challenged with the moderately virulent CSFV vALV-A76 strain at a dose of 10^6.0^ TCID_50_ and monitored for 14 days; dpv, days postvaccination; **antibody detection using immunochromatographic test strip; dpv, days postchallenge; SNT, serum neutralization test; −, negative; +, positive.

## 4 Discussion

Vaccination programs employing conventional CSFV LAVs have been demonstrated to protect pigs against CSFV infection and clinical signs in early-stage vaccination (Blome et al., 2006; Graham et al., 2012). Nevertheless, these conventional vaccines cannot resolve the challenges associated with the DIVA concept and the interference of MDAs. Various CSFV marker vaccines have been developed to address this challenge, such as subunit vaccines or chimeric pestiviruses designed to fulfill the DIVA strategy for controlling and eradicating CSF (Reimann et al., 2004; Gong et al., 2019; Lim et al., 2019; Park et al., 2021; Huynh et al., 2023). With the improvement of vaccine candidates, DIVA diagnostic tools using ELISA kits for detecting CSFV E^rns^ antibody or monitoring the induction of neutralizing antibodies using some commercial ELISA kits for E2 antibody detection have been developed and employed as adjunctive diagnostic assessments together with the E2 subunit vaccine and chimeric pestiviruses (Meyer et al., 2017; Wang et al., 2020). However, ELISA procedures are time-consuming and require skilled personnel, which might not be suitable for rapid field testing if E2 and E^rns^ antibody detection needs to be evaluated simultaneously with a large sample number in the field. Colloidal gold ICS have been established and are a valuable diagnostic tool for monitoring antibodies against CSFV in vaccinated pigs (Li et al., 2012; Bai et al., 2022; Xu et al., 2022). Compared to alternative antibody detection methodologies such as SNT or ELISA, ICS is notably user-friendly, rapid, and easily transportable (Li et al., 2012; Wang et al., 2020). Thus far, ICS development has preferably focused on detecting antibodies against the CSFV E2 protein, a powerful tool for the CSFV antibody assessment (Bai et al., 2022; Xu et al., 2022). Meanwhile, ICS development for CSFV E^rns^ antibody detection for the DIVA diagnostic approach still has no specific innovation (Wang et al., 2020). Furthermore, considering the benefits of transient protein expression in plant-derived systems (Yamamoto et al., 2018; Laughlin et al., 2019; Park et al., 2019), a novel ICS has been developed for the simultaneous detection of dual CSFV E2 and E^rns^ antibodies. This study established ICS based on colloidal gold recombinant E2 and E^rns^ proteins derived from *N. benthamiana* plants to rapidly evaluate CSFV antibodies and integrate DIVA diagnostic features.

In protein manipulation, previous studies have indicated that plant-derived transient expression enhances high protein expression and yield, immunogenicity in animal experiments, and high binding affinity for antibody detection absent in certain conventional expression systems (Sohn et al., 2018; Yamamoto et al., 2018; Xu et al., 2022). Compared to the transgenic approach for CSFV E2 protein expression using baculovirus (Wei et al., 2021; Bai et al., 2022), mammalian cells (Ji et al., 2018; Chen et al., 2023), or the *E. coli* system (Li et al., 2012; Yi et al., 2022), the CSFV E2-expressing transgenic plant-derived system was preferable in terms of a large amount and stable expression of the target protein and inexpensiveness, as in recent studies (Sohn et al., 2018; Laughlin et al., 2019; Park et al., 2019, 2020; Xu et al., 2022). This approach facilitated the purification of recombinant E2 from *N. benthamiana* extracts. Although the E2 protein is typically dimeric form, earlier investigations (Park et al., 2019) have confirmed a multimeric form of E2 protein expressed in plant tissue through glycosylation. A few systems have been established for recombinant E^rns^ protein expression in *E. coli* (Aebischer et al., 2013; Yi et al., 2022), baculovirus (Wei et al., 2021), or yeast (Huang et al., 2006; Luo et al., 2015), but there are several concerns regarding inappropriate posttranslational modifications, particularly the absence or low proportion of glycosylation in E^rns^ or E2 protein that may influence the binding affinity of antibodies (Gavrilov et al., 2011). The most important feature of CSFV E2 and E^rns^ is their heavy glycosylation, particularly E^rns^, which contains seven putative *N*-linked glycosylation sites. *N*-glycosylation results in a complex mixture of neutral and monosialylated multiantennary *N*-glycans, accounting for nearly half of the molecular mass of mature glycoproteins (Branza-Nichita et al., 2004; Gavrilov et al., 2011). Interestingly, in terms of *N*-glycosylation, a previous study (Strasser, 2023) reported that *N. benthamiana* plant-based expression platforms exhibit remarkable versatility in that they can effectively accommodate transient and stable engineering of the *N*-glycan processing pathways without inducing cell death or generating adverse growth phenotypes associated with biomass production, protein expression or yield, features lacking in conventional expression systems. In this study, protein expression using a plant-derived system was employed as a first report for E^rns^ protein expression in which the yielded E^rns^ protein had a molecular mass of ~45 kDa, comparable to the mature E^rns^ protein in previous studies (Thiel et al., 1991; Bhattacharya et al., 2019). Although it was speculated that E2 and E^rns^ proteins would be highly glycosylated in the plant expression system, the purified proteins were successfully conjugated with colloidal gold in this study to develop the ICS, thereby enhancing their availability.

This study collected various sera to assess the sensitivity and specificity of ICS and its correlation with commercial ELISA kits. As mentioned, ICS showed high sensitivity or specificity for detecting CSFV E2 and E^ms^ antibodies under different vaccination or infection backgrounds, particularly E^rns^ detection, even at low SNT titers (Tables 1, 5), which would be difficult to confirm through ELISA kits. The performance of ICS exhibited high coincidence rates with ELISA kits detecting E2 and E^ms^ antibodies. However, the preference for detecting E2 antibodies was evident, particularly later when the E2 subunit vaccine was employed in this study (Supplementary Table S1). Despite ICS exhibiting sensitivity with SNT titers of 16 with serum samples from pigs vaccinated with the modified live LOM strain (Table 1), it surprisingly demonstrated high sensitivity in detecting E2 antibodies in serum samples from pigs vaccinated with commercial conventional CSFV GPE^−^ LAV or chimeric vaccines, even at lower SNT titers of 16 (Tables 5, 6; Supplementary Table S2). Although this study confirmed high sensitivity and specificity across a diverse range of sera, the sample size was small. Therefore, future studies should employ larger sample sizes to assess the reproducibility of ICS. Given the impact of storage temperature, time, and manufacturing batches on the reliability of detection, further research is essential to evaluate the repeatability and stability of ICS. In addition, because the dependence of CSFV-specific antibody responses on several factors, such as CSFV infection with different genotypes, the induction of E2-or E^rns^-specific antibody (Popescu et al., 2019), and the failure of vaccination or the delay of antibody responses against CSFV induced by PRRSV (Chen et al., 2019; Li et al., 2020), a comprehensive approach involving a combination of various methods and continuous monitoring is essential to accurately determine the antibody response against CSFV.

ICS was used to evaluate the DIVA profiles of two chimeric pestiviruses, generated based on an infectious cDNA clone derived from the GPE^−^ LAV strain. These chimeric viruses possess the E^rns^ glycoprotein, which is phylogenetically distant from CSFV, and are potential DIVA vaccine candidates (Huynh et al., 2023). Serum samples obtained from optimal vaccination dose experiments and vaccine-efficient studies were reused to evaluate the DIVA profiles of these chimeric vaccine candidates. In the optimal vaccination dose experiment, successful detection of anti-CSFV E^rns^ antibodies in serum samples from conventionally vaccinated pigs was confirmed, whereas all chimeric-vaccinated pigs were negative, indicating the effectiveness of this ICS in detecting specific CSFV E^rns^ antibodies. Meanwhile, the presence of an E2-specific antibody indicated that antibody responses depended on the individual and the time point postvaccination (Supplementary Table S2). Significantly, the DIVA capability of chimeric vaccine candidates was validated in vaccine efficacy studies focusing on CSFV E^rns^ detection, by which ICS successfully distinguished chimeric-vaccinated animals from infected and conventionally vaccinated animals. The outcomes obtained provide valuable insights into the potential of this ICS to address the limitations of previous ICS that lack DIVA diagnostic capabilities with E^rns^ antibody detection (Li et al., 2012; Bai et al., 2022; Xu et al., 2022). Although CSFV E^rns^ is used as a negative marker to evaluate the feasibility of ICS for DIVA in this study, future studies should concentrate on developing a serological antibody assay capable of detecting antibodies against recombinant PAPeV E^rns^ and PhoPeV E^rns^. This development will facilitate the implementation of marker vaccine candidates vGPE^−^/PAPeV E^rns^ and vGPE^−^/PhoPeV E^rns^ to control and eradicate CSF in the future. In addition, DIVA application with E^rns^ antibody detection was not feasible to evaluate CSF-free status individually in animals, a limitation of the sera used in this study. Instead, detection of E^rns^ CSFV-specific antibodies was suggested for implementation at the herd level, as validated in previous studies (Schroeder et al., 2012; Pannhorst et al., 2015). Therefore, further investigations are necessary to comprehensively validate ICS specificity and sensitivity across larger populations under various field conditions. Because serum samples derived from CSFV genotype 1 were mainly used in this study, evaluating its performance against diverse strains, genotypes, and its potential cross-reactivity with antibodies from other swine pathogens in further studies will enhance its reliability and applicability in practical DIVA strategies.

Until now, the gold standard for determining antibodies against CSFV is SNT. However, existing commercial ELISA kits evaluate E2 or E^rns^ separately, which incurs additional costs and labor and is more time-consuming. Although ICS development for antibody detection has been established, previous efforts have primarily focused on detecting CSFV E2 protein antibodies. This study is a pioneering effort to develop an ICS capable of detecting E2 and E^rns^ antibodies simultaneously. This ICS addresses the prior limitation of being unable to differentiate infected from vaccinated animals from previous studies (Bai et al., 2022; Xu et al., 2022). Extensive studies and long-term monitoring are also essential to evaluate the DIVA potential of subunit or chimeric vaccines that contribute significantly to CSF surveillance and control.

## 5 Conclusion

E2 and E^rns^ and antigens derived from the plant showed great potential and can be used to engineer a CSFV E2/E^rns^ dual-antibody ICS, which is suitable for application in the field to monitor CSFV antibodies in vaccinated pig herds. ICS not only demonstrated high correlation with commercial ELISA kits but also exhibited sensitivity and specificity for detecting E2 and E^rns^ antibodies. Thus, ICS is a rapid diagnostic tool for detecting antibodies against CSFV. The most vital point of ICS developed in this study is that it can satisfy the DIVA concept using two chimeric vaccine candidates, vGPE^−^/PAPeV E^rns^ and vGPE^−^/PhoPeV E^rns^. Future work should be conducted to evaluate the stability of ICS and verify its repeatability.

## Data availability statement

The datasets presented in this study can be found in online repositories. The names of the repository/repositories and accession number(s) can be found in the article/Supplementary material.

## Ethics statement

The animal study was approved by the Institutional Animal Care and Use Committee of the Faculty of Veterinary Medicine, Hokkaido University (approval no. 18-0038, approved on March 26, 2018, and approval no. 23-0029, approved on March 23, 2023) and performed according to the guidelines of this committee. The genetic modification experiments were performed according to the Cartagena Protocol on Biosafety under the Convention on Biological Diversity and approved by the Institutional Committee on the Safety of Genetic Modification Experiments of Hokkaido University and the Ministry of Education, Culture, Sports, Science and Technology (approval no. 2020-009 and 5-Monkashin-Dai-479, approved on June 8, 2021 and August 31, 2023, respectively). The study was conducted in accordance with the local legislation and institutional requirements.

## Author contributions

LH: Conceptualization, Data curation, Formal analysis, Investigation, Methodology, Validation, Visualization, Writing – original draft, Writing – review & editing. E-JS: Conceptualization, Data curation, Investigation, Methodology, Project administration, Resources, Supervision, Validation, Writing – review & editing. YP: Conceptualization, Data curation, Investigation, Methodology, Writing – review & editing. JK: Conceptualization, Data curation, Investigation, Methodology, Writing – review & editing. TS: Data curation, Investigation, Writing – review & editing. TH: Data curation, Investigation, Writing – review & editing. NI: Data curation, Investigation, Writing – review & editing. S-HH: Data curation, Investigation, Writing – review & editing. H-NL: Data curation, Investigation, Writing – review & editing. YS: Conceptualization, Data curation, Investigation, Methodology, Project administration, Resources, Supervision, Validation, Writing – review & editing.

## Footnotes

^1^ https://zendto.bioneer.co.kr/codon/index.py
