# Supplementary material for: Development of a dual immunochromatographic test strip to detect E2 and Erns antibodies against classical swine fever
Source: Front Microbiol. 2024 Apr 11;15:1383976. doi: 10.3389/fmicb.2024.1383976 (PMC11043574; doi:10.3389/fmicb.2024.1383976)
Supplement: Supplementary file 1 [file Data_Sheet_1.pdf]

# *Supplementary Material*

## 1 Supplementary Figures and Tables

### 1.1 Supplementary Figures

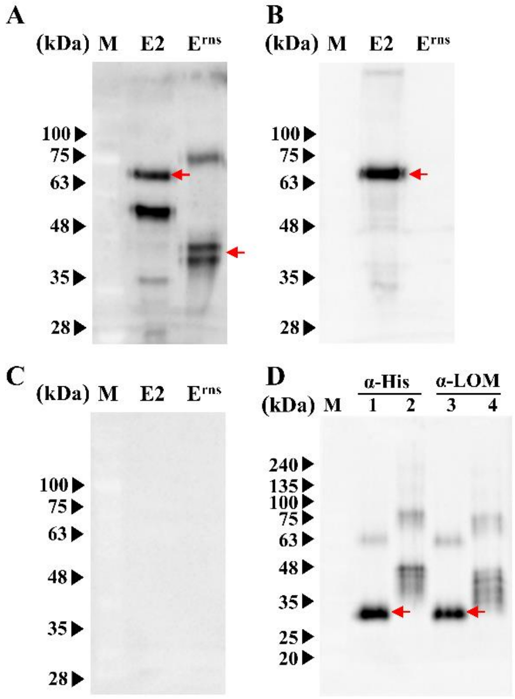


**Supplementary Figure S1.** Expression and identification of CSFV E2 or $E^{rns}$ protein. (A) Western blot analysis confirmed the recombinant CSFV E2 or $E^{rns}$ protein using anti-LOM vaccinated serum. (B) The recombinant CSFV E2 or $E^{rns}$ protein was confirmed in Western blotting using anti-E2 subunit vaccinated serum. C) Western blotting using anti-native porcine serum as negative control. (D) Western blot analysis of purified $E^{rns}$ protein using anti-His antibody for lane 1 (treated with PNGase F), and lane 2 (without PNGase F), or using anti-LOM vaccinated serum for lane 3 (treated with PNGase F) and lane 4 (without PNGase F). M, protein size marker. Red arrows represent the expression of E2 or $E^{rns}$ protein.

## 1.2 Supplementary Tables

**Supplementary Table S1**. Detection of E2 and $E^{rns}$ antibodies using ELISA and immunochromatographic test strip in vaccinated pigs.

| Pig ID | Antibody detection | | | | | | | | | | | | | | | | | |
|---|---|---|---|---|---|---|---|---|---|---|---|---|---|---|---|---|---|---|
| | E2 | | | | | | | | | $E^{rns}$ | | | | | | | | |
| | 0 dpv* | | | 20 dpv** | | | 40 dpv | | | 0 dpv* | | | 20 dpv** | | | 40 dpv | | |
| | Com 1 | Com 2 | ICS | Com 1 | Com 2 | ICS | Com 1 | Com 2 | ICS | Com 3 | Com 4 | ICS | Com 3 | Com 4 | ICS | Com 3 | Com 4 | ICS |
| #1 | – | – | – | + | – | – | + | + | + | – | – | – | – | – | – | – | – | – |
| #2 | – | – | – | + | – | – | + | + | + | – | – | – | – | – | – | – | – | – |
| #3 | – | – | – | + | – | – | + | + | + | – | – | – | – | – | – | – | – | – |
| #4 | – | – | – | + | – | + | + | + | + | – | – | – | – | – | – | – | – | – |
| #5 | – | – | – | + | – | + | + | + | + | – | – | – | – | – | – | – | – | – |
| #6 | – | – | – | + | – | – | + | + | + | – | – | – | – | – | – | – | – | – |

*First vaccination shoot; **booster shot; dpv: days postvaccination; Com 1, commercial Bionote ELISA kit; Com 2, commercial IDEXX ELISA kit; Com 3, commercial Indical Bioscience ELISA kit; Com 4, commercial Thermo Fisher Scientific ELISA kit; ICS, immunochromatographic test strip; –, negative; +, positive.

**Supplementary Table S2.** Detection of E2 and $E^{rns}$ antibody responses from serum samples in pigs post inoculated with the commercial vaccine CSFV strain $GPE^{-}$ at different time points using the immunochromatographic test strip and serum neutralization test.

| Pig ID | Days postvaccination | Antibody detection using ICS | | SNT titer |
|---|---|---|---|---|
| | | E2 | $E^{rns}$ | |
| #135 | 0 | – | – | 2※ |
| | 45 | + | + | 128 |
| | 90 | + | + | 64 |
| | Before slaughtering | + | + | 128 |
| #139 | 0 | – | – | 2※ |
| | 45 | + | + | 128 |
| | 90 | + | + | 256 |
| | Before slaughtering | + | + | 512 |
| #168 | 0 | + | – | 32※ |
| | 45 | – | + | 16 |
| | 90 | + | + | 16 |
| | Before slaughtering | + | + | 16 |
| #179 | 0 | + | + | 32※ |
| | 45 | – | + | 4 |
| | 90 | + | + | 4 |
| | Before slaughtering | + | + | 16 |
| #182 | 0 | + | – | 32※ |
| | 45 | – | – | 8 |
| | 90 | + | – | 16 |
| | Before slaughtering | + | – | 16 |
| #190 | 0 | + | – | 64※ |
| | 45 | – | – | 16 |
| | 90 | + | + | 32 |
| | Before slaughtering | + | – | 16 |

※Maternally derived antibodies; SNT, serum neutralization test; ICS, immunochromatographic test strip; –, negative; +, positive.
